# Pediatric ultrasound practice in Italy: an exploratory survey

**DOI:** 10.1186/s13052-024-01680-3

**Published:** 2024-06-09

**Authors:** Anna Maria Musolino, Monica Tei, Cristina De Rose, Danilo Buonsenso, Maria Chiara Supino, Stefania Zampogna, Annamaria Staiano, Massimiliano Raponi, Antonella Amendolea, Vincenzo Colacino, Laura Gori, Alessandro Manganaro, Riccardo Ricci, Victoria D’Inzeo, Salvatore Grosso, Alberto Villani, Rino Agostiniani

**Affiliations:** 1 Unit of Emergency Pediatrics, Department of Emergency, Admission and General Pediatrics, Bambino Gesù Children’s Hospital (IRCCS), Rome, Italy; 2 Clinical Pediatrics, Department of Mother and Child, Siena University Hospital, Viale Bracci 16, Siena, 53100 Italy; 3 Department of Woman and Child Health and Public Health, Fondazione Policlinico Universitario Agostino Gemelli IRCCS, Rome, Italy; 4 Department Pediatrics, Hospital of Crotone President of SIMEUP (Italian Society of Pediatric Emergency Medicine Urgency), Crotone, Italy; 5 Department of Translational Medical Sciences, Section of Pediatrics, University of Naples “Federico II”, President of SIP (Italian Society of Pediatric), Naples, Italy; 6 Management and Diagnostic Innovations and Clinical Pathways Research Area, Medical Directorate, Bambino Gesù Children’s Hospital, IRCCS, Rome, 00165 Italy; 7 Pediatric Unit, Cecina Civil Hospital, Cecina, Italy; 8 Health District of Central Friuli, Cividale del Friuli, Italy; 9 Department of Maternal and Child Health, Santa Chiara Hospital, University of Pisa, Pisa, 56100 Italy; 10 Pathology and Neonatal and Pediatric Intensive Care Unit, University Hospital G. Martino, Messina, Italy; 11 Professional Development, Continuing Education and Research, Bambino Gesù Children’s Hospital (IRCCS), Rome, Italy; 12 Department of Cardiac Surgery, Cardiology and Heart and Lung Transplant, Bambino Gesù Children’s Hospital (IRCCS), Rome, Italy; 13 Unit of General Pediatrics, Department of Emergency, Admission and General Pediatrics, Bambino Gesù Children’s Hospital (IRCCS), Rome, Italy; 14 Department of Pediatrics and Neonatology, San Jacopo Hospital, Via Ciliegiole 97, 51100 Pistoia, Italy

**Keywords:** Pediatric ultrasound, Education, Training, Pediatric residency, Point-of-care ultrasound, Survey

## Abstract

**Background:**

The aim of this exploratory survey is to describe the current state of US (ultrasound) technique across different pediatric settings nationwide.

**Methods:**

A questionnaire was emailed to all members of the Italian Society of Pediatrics, including pediatric residents. The survey was open from December 2021 to March 2022.

**Results:**

There were 1098 respondents. Seven hundred and seven pediatricians (84.1%) reported any use of US, while 51 (44.3%) residents denied it. The majority of participants (*n* = 956, 87.1%) reported to have a US machine available within the department, mostly cart-based (*n* = 516, 66.9%) and provided from 1 to 5 years prior to the survey (*n* = 330, 42.8%). Lung and neonatal cerebral regions were the most frequently scanned (*n* = 289, 18.7% and *n* = 218, 14.1%, respectively). The suspicion of pneumonia or respiratory distress represented the main reasons for performing US in emergency room (*n* = 390, 78% and *n* = 330, 66%, respectively). The majority of family pediatricians reported to scan lung and kidney/urinary tract regions (*n* = 30, 16.9%, and *n* = 23,12.9%, respectively). Regarding US training, the majority of respondents (*n* = 358, 34.6%) declared an experience-based education, with a deficient certification enabling the use of US in 71.6% (*n* = 552) of cases. The most common barriers included the lack of a well-defined training program (*n* = 627, 57.1%), unavailability of the US machine (*n* = 196, 17.9%) and legal responsibility concern (*n* = 175, 15.9%).

**Conclusions:**

Despite the growing interest on pediatric US nationally, significant barriers still limit widespread adoption. These obstacles may be addressed through the dissemination of a specific US education plan and providing additional resources.

**Supplementary Information:**

The online version contains supplementary material available at 10.1186/s13052-024-01680-3.

## Introduction

The growing interest toward ultrasound (US) in recent years is basically due to a non-invasive and portable nature of this imaging tool [1]. Being free from ionizing radiation and painless, US is suitable also for neonatal and pediatric age, with a wide range of applications supported by research literature [2]. Pediatric consultants are becoming more and more self-confident in US practice, which is becoming integral to the physical examination and may help physicians for decision-making process, follow up management of acute diseases and therapeutic procedures [3, 4].

Despite a remarkable body of evidence, a routine use of US in pediatrics cannot be recognized yet [5, 6]. Moreover, literature investigating US application almost entirely relies on the emergency and critical medicine experience [7] while deficient data exist for its use in different pediatric settings, such as non-intensive pediatric units or outpatient clinics.

Significant gaps still need to be addressed in order to implement US dissemination. To date, the lack of core infrastructural elements is one of the most perceived barrier in US widespread development. Different practice environments may affect the limitation size even if literature is controversial. Some authors report that divisions with larger units invest more resources for technology, have an adequate number of faculty and a greater possibility of collaborating with specialists (i.e., cardiology, radiology) [8, 9]. Conversely, Conlon et al. found that limited access to US machines is independent of the division size [10]. The lack of or inadequate diagnostic imaging equipment is reported as a major barrier for US implementation also in low- and middle-income countries, as underlined in a recent sistematic review [11]. Definitely, the availability of US equipment and machines throughout various clinical settings should be enhanced, also favoring the use of portable and handheld devices with lower cost but good resolution [12, 13].

Nevertheless, the US application is limited by another main factor that is education. Although US has become a powerful tool for treating clinicians, it is dependent on the user’s skills and training. A great push towards the importance of training and standardized educational curriculum has been made through national guidelines publications [14, 15]. Despite this, at present, a standardization of training pathways for both pediatric residents and faculty has not been established and US curricula and credentialing processes deeply vary worldwide [10, 16, 17]. Furthermore, even if a brief educational intervention has been demonstrated to be effective in increasing proficiency on US [12], the lack of learning time as well as the paucity of skilled trainers are also reported limitations in literature [16, 18]. As the use of US continues to increase, the need for early training also increases: numerous pieces of evidence report how starting upstream training from the medical school period would ease US application postgraduating [19–21].

The objective of this national survey is to investigate the use of US among pediatricians and pediatric residents and to describe the characteristics of the US machines, the number of scans carried out, the years of experience of the performers and the main US applications.

across different pediatric settings. Another main purpose of the survey is to identify training needs and possible barriers to US implementation.

## Materials and methods

The present study is a national, cross-sectional, web-based survey. The questionnaire was emailed to all members of the Italian Society of Pediatrics, including pediatric residents and it was distributed for completion from December, 2021 to March, 2022.In order to optimize the return rate of the survey, reminder e-mails were sent 3 times during the period and the subsequent analysis of the results was performed only for surveys which were fully completed one month after the third reminder. Written consent was not required due to the anonymous and voluntary nature of the questionnaire. Ethical consent was not required due to the study design and local regulations. The estimated time to complete the questionnaire was 6 min.

The questionnaire ranged from24 to 27 items, depending on whether pediatricians or residents answered, respectively. The full version of the survey is reported as annex.

The questions were formulated as multiple-choice response, with some exceptions (four-point Likert scales and open questions). For questions with “other” category, a free-text response was solicited.

Data were extracted from the SurveyMonkey platform and statistics was performed using IBM SPSS for Windows (Version 24.0, IBM Corp ).

## Results

The survey was completed by 1098 respondents with an overall response rate of 11%. Descriptive characteristics of survey respondents are reported in Table 1.

**Table 1:** Characteristics of Surveyed Participants

Characteristics of Participants, *n* (%)						
Age in years						
	< 30	31–40	41–50	51–60	> 60	To *n*(%)
**Gender**						
Female Male	107 (9,7) 35 (3,2)	194 (17,6) 68 (6,2)	157 (14,3) 58 (5,3)	143 (13,0) 76 (6,9)	109 (9,9) 151 (13,8)	710 (64,7) 388 (35,3)
**Current position**						
Resident Pediatrician Other^§^	140 (12,8) 2 (0,2) 0	48 (4,4) 214 (19,4) 0	2 (0,2) 211 (19,7) 2 (0,2)	2 (0,2) 217 (19,6) 0	6 (0,5) 254 (23,0) 0	198 (18) 898 (81,8) 2 (0,2)
**Region of Italy**						
North Centre South	17 (1,5) 81 (7,4) 44 (4,0)	84 (7,6) 93 (8,5) 85 (7,7)	71 (6,5) 76 (6,9) 68 (6,2)	44 (4,0) 87 (7,9) 88 (8,0)	46 (4,2) 102 (9,3) 112 (10,2)	262 (23,9) 439 (40,0) 397 (36,1)
**Setting**						
University/ teaching Hospital Private Hospital Community/District Hospital Family pediatrician Private practice	66 (6,0) 1 (0,1) 75 (6,8) 0 0	13 (1,2) 9 (0,8) 181 (16,5) 40 (3,6) 19 (1,7)	10 (0,9) 7 (0,6) 163 (14,8) 32 (2,9) 3 (0,3)	6 (0,5) 14 (1,7) 166 (15,1) 27 (2,4) 6 (0,5)	4 (0,4) 38 (3,5) 107 (9,7) 87 (7,9) 24 (2,2)	99 (9,0) 69 (6,4) 692 (63,0) 186 (16,9) 52 (4,7)
Department*						
General pediatric unit Pediatric ED Neonatology unit Adult ED Outpatient clinic Other^†^	60 (5,0) 41 (3,4) 40 (3,3) 0 1 (0,1) 0	85 (7,0) 81 (6,7) 92 (7,6) 25 (2,0) 44 (3,6) 2 (0,2)	79 (6,5) 40 (3,3) 73 (6,0) 13 (1,1) 40 (3,3) 35 (2,9)	76 (6,3) 39 (3,2) 70 (5,8) 19 (1,5) 34 (2,8) 39 (3,2)	53 (4,4) 13 (1,1) 50 (4,1) 10 (0,8) 26 (2,1) 28 (2,3)	353 (29,2) 214 (17,7) 325 (26,9) 67 (5,5) 145 (12) 104 (8,6)

*Some respondents reported multiple settings

§Other: 2 radiologists

†Other: 52 private practice, 18 neonatal intensive care unit, 14 pediatric intensive care unit, 14 district outpatients clinic and 6 on retirement

ED: emergency department

**Fig. 1:**
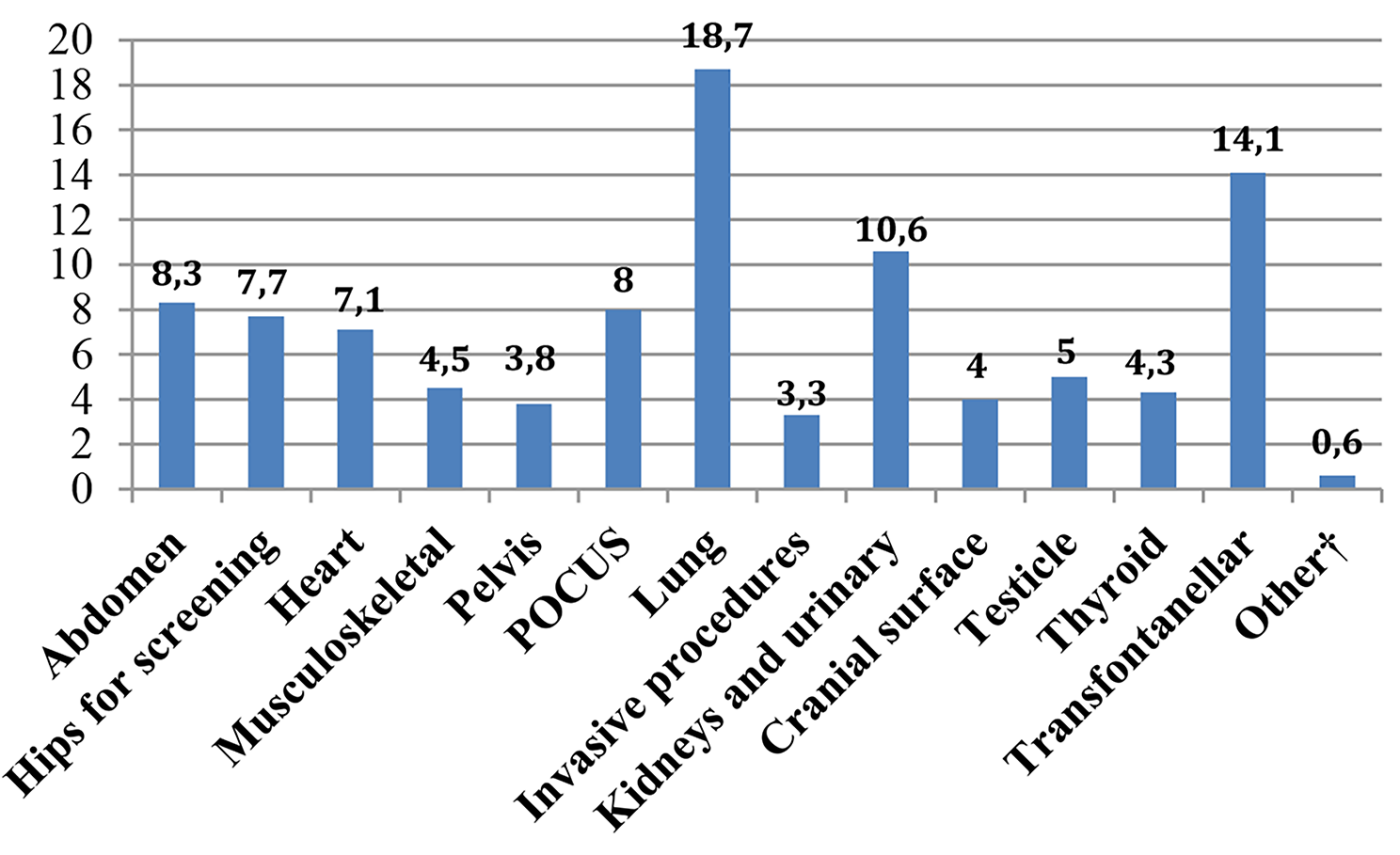
US Applications. Legend: Data are expressed as a percentage. †Other: ocular and female reproductive system US

**Fig. 2:**
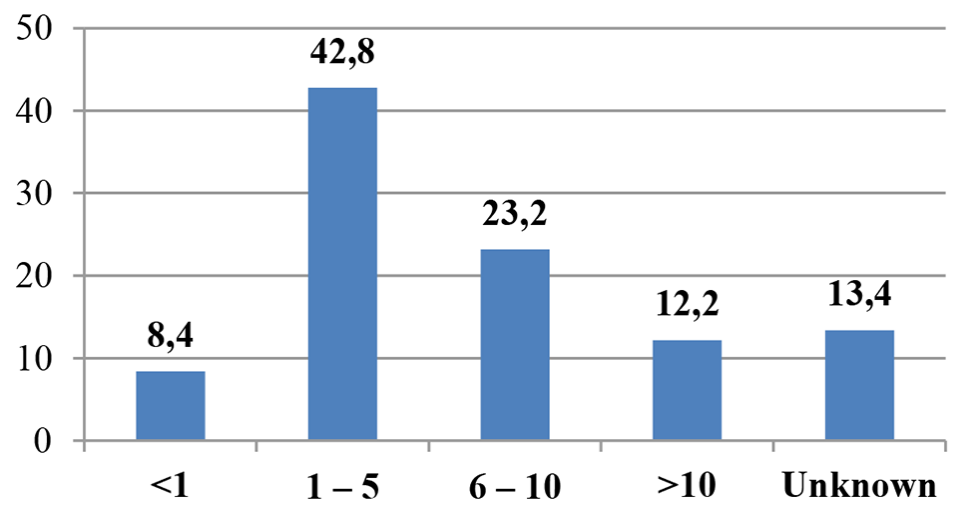
When US machine in use at time of the survey was provided (years). Legend: Data are expressed as a percentage

**Fig. 3:**
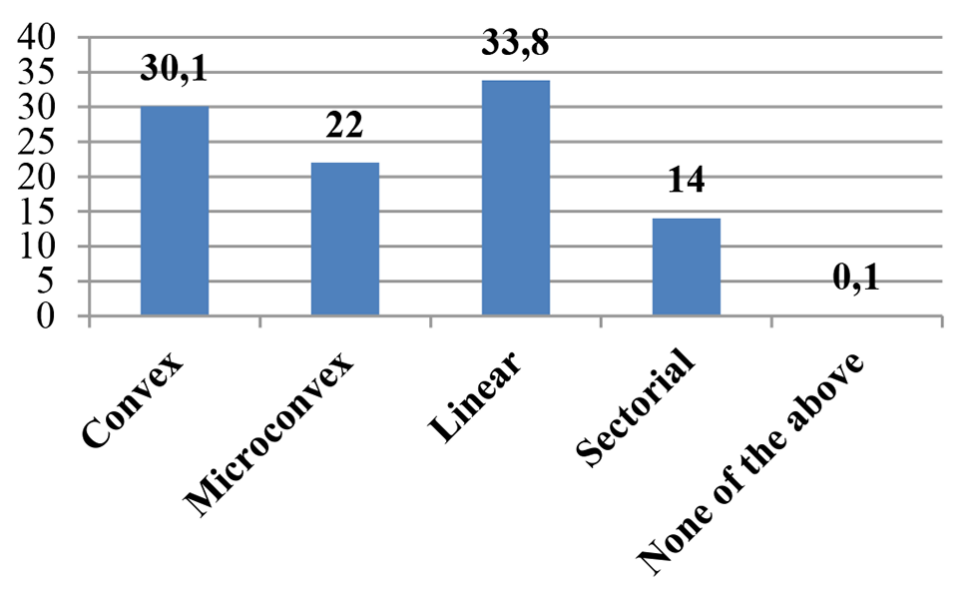
Type of probe available. Legend: Data are expressed as a percentage. Respondents reported multiple answers

Most of participants (87.1%, *n* = 956) declared to have an US machine available within the department. The predominant model resulted to be cart-based (66.9%, *n* = 516), followed by portable (28.5%, *n* = 220) and handheld (4.5%, *n* = 35). Linear and convex were the most available probes (33.8%, *n* = 551 and 30.1%, *n* = 491,respectively). Nearly one third of respondents (42.8%, *n* = 330) assessed that US machines had been provided from 1 to 5 years prior to the survey while only 12.2% (*n* = 94) from more than 10 years. Lung and neonatal cerebral US were the most frequently performed by participants (18.7%, *n* = 289 and 14.1%, *n* = 218, respectively). Details of US applications are reported in Figs. 1, 2 and 3.

Regarding US utilization, 707 (84.1%) pediatricians reported any use of US in clinical practice while 51 (44.3%) residents denied it. Among pediatric residents, 71.8% (*n* = 46) asserted to use US with less than one year of experience. Moreover, more than half residents(59.4%, *n* = 38) declared a frequency of scans less than 10 per month. On the other hand, 36.4% of pediatricians (*n* = 257) stated to have more than ten-years experience and 46% (*n* = 325) referred to perform more then twenty exams per month.

Personal position and expertise about US utilization and differences between university/not university and public/private settings are summarized in Tables 2, 3 and 4.

**Table 2:** Personal position and expertise about US application for pediatricians and residents

Personal position towards US application		Pediatricians	Residents	Tot *n* (%)
Use of US				
	Yes	707 (84.1)	64 (55.7)	771 (80.6)
	No	134 (15.9)	51 (44.3)	185 (19.4)
Experience in US (years)				
	< 1	89 (12.6)	46 (71.8)	135 (17.5)
	2–5	243 (34.4)	12 (18.8)	255 (33.1)
	6–10	118 (16.7)	0 (0)	118 (15.3)
	> 10	257 (36.4)	6 (9.4)	263 (34.1)
Number of scans in a month				
	< 10	202 (28.6)	38 (59.4)	240 (13.1)
	11–20	180 (25.5)	16 (25)	196 (25.4)
	> 20	325 (46)	10 (15.6)	335 (43.5)

**Table 3:** Personal position and expertise about US application in university and not university setting

Personal position towards US application		University	Not University	Tot *n* (%)	*p*
Use of US					
	Yes	87 (82,8)	684 (80,4)	771 (80.6)	Ns
	No	18 (17,2)	167 (19,6)	185 (19.4)	
Experience in US(years)					
	< 1	10 (11,5)	125 (18,3)	135 (17.5)	
	2–5	34 (39,1)	221 (32,3)	255 (33.1)	Ns
	6–10	6 (6,9)	112 (16,4)	118 (15.3)	
	> 10	37 (42,5)	226 (33)	263 (34.1)	
Number of scans in a month					
	< 10	13 (14,9)	227 (33,2)	240 (13.1)	
	11–20	36 (41,4)	160 (23,4)	196 (25.4)	Ns
	> 20	38 (43,7)	297 (43,4)	335 (43.5)	

**Table 4:** Personal position and expertise about US application in public or private setting

Personal position towards US application		Public	Private	Tot *n* (%)	*p*
Use of US					
	Yes	682 (79,8)	89 (88,1)	771 (80,6)	Ns
	No	173 (20,2)	12 (11,9)	185 (19,4)	
Experience in US (years)					
	< 1	129 (18,9)	6 (6,7)	135 (17,5)	
	2–5	247 (36,2)	8 (9,1)	255 (33,1)	Ns
	6–10	104 (15,3)	15 (16,8)	118 (15,3)	
	> 10	202 (29,6)	38 (42,7)	263 (34,1)	
Number of scans in a month			36 (40,5)		
	< 10	225 (33)	15 (16,8)	240 (31,1)	
	11–20	158 (23,2)	38 (42,7)	196 (25,4)	Ns
	> 20	299 (43,8)	36 (40,5)	335 (43,5)	

There were no statistically significant differences in the use of US by dividing the sample by working department and geographical area (north, central or south).

More than half respondents (55.7%, *n* = 430) affirmed to use ultrasounds in the emergency room, for most cases in the suspicion of pneumonia (78%, *n* = 390), or for patients with respiratory distress (66%, *n* = 330). 44% (*n* = 360) of participants used ultrasounds for trauma cases while 38% (*n* = 311) for undifferentiated shock Among family pediatricians, 177 (95.2%) declared to use US routinely, mainly for lung and kidney/urinary tract regions (16.9%,*n* = 30 and 12.9%, *n* = 23, respectively).

With regard to US training and certification, data are reported in Table 5.

**Table 5:** US training and certification

US training and certification		Pediatricians	Residents	Tot *n* (%)
US training				
	Lectures with practical session	271 (28.6)	20 (23)	291‡ (28.1)
	Advanced training course	203 (21.4)	10 (11.5)	213‡ (20.6)
	Online course	118 (12.4)	13 (14.9)	131‡ (12.7)
	On-site experience	320 (33.8)	38 (43.7)	358‡ (34.6)
	Other^†^	36 (3.8)	6 (6.9)	42‡ (4)
Do you have a certification for echography use?				
	Yes	207 (29.3)	12 (18.8)	219 (28.4)
	No	500 (70.7)	52 (81.2)	552 (71.6)

‡Respondents reported multiple answers

^†^Other: simulation, integrated conferences, utilization of online resources, radiology unit attendance, fellowship in specialized center (in Italy or abroad)

In addition, 59.4% (*n* = 38) of residents claimed that US practice was not included in the training program while 94% (*n* = 36) of them considered desirable the insertion of a specific course during the residency.

**Fig. 4:**
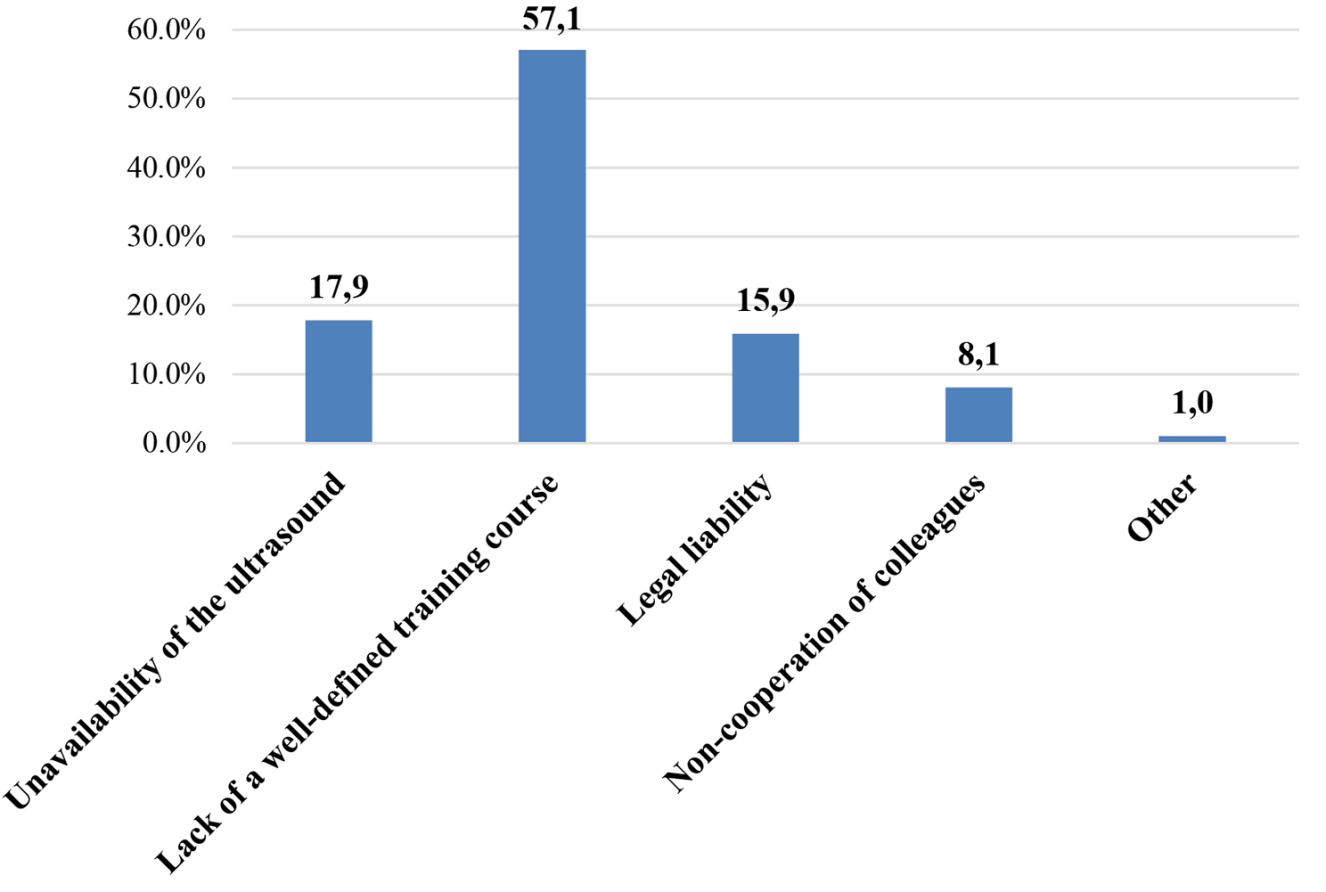
Limitations to pediatric US application. Legend: Data are expressed as a percentage

When asked to express about the main difficulties in using US, most participants complained a lack of a well-defined training program (57.1%, *n* = 627), 17.9%(*n* = 196) unavailability of the US machine, 15.9% (*n* = 175) legal responsibility concern, 8.1% (*n* = 89) non-collaboration of colleagues. In “other” category (1%, *n* = 11) participants listed as possible obstacles also insufficient educational time for learning US, lack of trained faculty to rely on and resistance to pediatric US application from other departments such as radiology or surgery (Fig. 4).

Almost all participants (98%, *n* = 1076) considered US a useful tool for clinical practice and they deemed US very or quite relevant in guiding clinical decisions (83.5%, *n* = 644 and 15.7%, *n* = 121, respectively).

169 (60.1%) of pediatricians working in ED have increased the use of ultrasound during the SarsCoV2 pandemic compared to 26.4% (216) of those working in other departments (*p* = 0.001). 153 (54.4%) of pediatricians working in ED have increased their knowledge during the COVID period compared to 111 (13.6% ) of those working in other departments (*p* = 0.001).

## Discussion

Since the use of US has increased exponentially worldwide in the last few decades, also for the pediatric age [1], we aimed to provide data on the current Italian state of pediatric US practice.

### US equipment and applications

The US spread is strictly related to the availability of machines: the majority of participants declared to have a US machine available for use within the department, with percentages in line with the literature [22, 23]. Particularly, convex and linear probes have been found to be the ones most obtainable for US examinations.

US practice seems to be relatively recent tool in pediatrics [4], considering that machines have been provided mostly 1–5 years prior to the survey. Nevertheless, the vast majority of US machines have been found to be conventionally cart-based, at the expense of newer models such as portable and handheld systems [13], which have been demonstrated to improve patient outcomes also in environments with limited resources [24]. These results focus on the urgent need to direct more resources for improving US equipment and advanced scanning technologies in pediatric units.

Concerning the type of examination, lung resulted to be the most common scanned region. We also found that participants working in the emergency room, mostly use US in suspicion of lung pathologies. Similar results were also highlighted for family pediatricians. A possible explanation for this finding may be the steep learning curve which also allows novices to be able to perform lung US [25]. It should be also underlined that nearly one third of respondents assessed to use US for cardiac and abdominal application: traditionally, the use of US for these two anatomic regions is a prerogative of cardiologists and radiologists, respectively [5]. This finding may be considered as the beginning of a growth path to be pursued in the future for the entire pediatric personnel [26, 27].

### US practice among different pediatric settings

Some differences on US practice between pediatric residents and pediatricians have been found in this survey. Among residents, only half declared to use US, mostly with little experience and low scanning frequency. On the contrary, the majority of attending pediatricians routinely perform US, although less than half of them with experience over ten years and a scanning frequency more than twenty per month. Controversial data are reported in literature about disparity in US application between residents and pediatricians [6]: regarding the Italian situation, we can speculate that, despite the growing interest on US in last decades, the method still needs to be spread and implemented in clinical practice, especially during residency.

The vast majority of the surveyed family pediatricians report to perform US routinely. As far as we know, the present survey is the first study investigating US application in pediatric family care, so we are not able to compare our data with similar findings in literature. Our results even seem to be in contrast with those described by a recent survey which reports a low percentage of US scans performed among family medicine residents and practicing physicians [28]. We hypothesize that the high percentage of US utilization among family pediatricians may reflect a bias selection.

Finally, we did not find statistically significant differences for US practice dividing the sample by geographical area (north, central or south) or working department. We definitely believe that this last result should be verified on a larger study sample. We were not even able to compare our data with those in literature since, as far as we know, there are no studies comparing the US use in the private, public or university setting nor for different geographical areas of the same nation.

### US and education

The section of the survey dedicated to training underlined interesting issues. To date, an informal experience-based training or theoretical-practical courses have been found to be the most frequently adopted while a credentialing process was missing in most cases. Similar findings are reported in literature, pointing out the lack of specific training pathways and non-homogeneous programs [29–31]. Learning methods also vary based on different realities. In middle and low-income countries, e-learning methods have been reported to be the commonest method, related to the high costs of face-to face training [11]. Nevertheless challenges relating to poor internet connectivity still affects access to study platform and communication with supervisors [11].

The standardization of training plans, starting preferably during residency rather than early under graduation medical period, might allow to create a US curriculum as guarantee of educational pathway and quality assessment [32, 33]. Furthermore medical school/residency directors and their institutions should consider the curriculum as a core requirement for the implementation of US technique [33]. More than half respondents within the pediatric residents subgroup declared that a specific training program has yet to be described; however, almost all of them showed a positive attitude towards US, supporting its endorsement during residency, as previously underlined in literature [5, 34].

### Barriers to POCUS applications

Our survey points out possible barriers to POCUS applications. Among these barriers, the lack of well-defined training plan has been identified as a main obstacle to US expansion by respondents: we strongly believe that an educational US curriculum should be mandatory not only for residents but also for attending physicians, as reported in recent literature [35, 36].

Despite the dramatic rise of US use, it is noteworthy that the unavailability of US machine is still considered as a barrier for daily practice in both our study and literature [8, 9]. Actually, some major reasons for complaints from physicians are the expensiveness of US machines and the subsequent difficulty in affording to purchase them by institutions, the scarce use of low-cost ultraportable devices, the US equipment deficiency and the inaccessibility to US machines for bedside use, often due to resistance from other departments [5, 8, 9, 30, 37]. The lack of technological devices for performing US is reported to be a barrier to the implementation of the method even in middle and low-income countries where clinicians often deal with the high costs of equipments, adverse climatic conditions, power instability, and inadequate maintenance service [11].

If the unavailability of US machines is indeed an absolute requirement for US practice, proper education still remains a main issue to be acknowledged [15]. First of all, sufficient time for training should be given since a number of reports indicates a lack of time to learn [9, 16, 30]. Moreover, a well-structured educational program could implement the small number of trainers within various institutions [8]. Last but not least, an ongoing education should be mandatory for all credentialed US physicians [15] in order to improve and consolidate their sonographic skills.

Regarding the liability concerns, they may be closely related to the lack of credentialing plan and quality assurance program in case of misinterpretation or misdiagnosis leading to malpractice claims [29, 38]. Again, healthcare professionals should be aware that literature provides non-apprehensive data about litigation directly related to US application in the last decade [39]. Furthermore, to improve ultrasound skill and reduce concerns about the sense of responsibility, with the possibility of using US more in daily clinical practice, it is not only necessary to train ultrasound skills but also training to imaging evaluation starting, for example, from the study of CT findings or anatomical information.

From the perspective of survey respondents, US has been highly scored in term of usefulness in order to integrate the patient’s clinical evaluation and guide clinical decision, confirming positive attitude toward US application [6, 12, 40, 41].

### POCUS during the COVID-19 pandemic

The last two questions of the survey investigated the perception of US role during the outbreak of the COVID19 pandemic. Italy has been the first european country to deal with COVID-19, serving a resilient assistance both in emergency departments and outpatient clinics nationwide [42]. Despite the enormous efforts to better understand clinical features of COVID-19 disease [42, 43], participants assumed that the pandemic does not seem to have implemented neither the use nor the knowledge expansion of US in pediatrics. This unexpected issue may be due to possible challenges in logistics of US examination (e.g. lack of portable ultrasound machines, high risk for contracting the COVID-19 infection) [44]. On the other hand, the further analysis for the subgroup of pediatricians working in ED found that both US use and knowledge were improved during the pandemic maybe due to a focused attention on emergency and COVID patients.

### Limitations and strenghts

Our study had a number of limitations and strenghts. The first limitation is intrinsic to the nature of the survey which is self-reported designed. Another limitation low response rate, which may reflect a lack of participant involvement and motivation on this topic. Actually, a potential response-bias could be due to the overestimation of US users among respondents, as those more interested in US technique may have been more like to respond. Regarding possible strengths, this study is one of the few survey which investigated the US application not only in pediatric emergency department but also in outpatient clinics. Moreover the survey was not targeting to a specific population sample, including pediatric residents, pediatric hospitalists and family pediatricians.

## Conclusions

This survey underlines a striking interest towards pediatric US for both pediatricians and residents nationwide. Pediatric residents support US training within the residency period in order to improve knowledge and confidence on the method. Nevertheless, the technique still needs to be implemented so that everyone can easily access it. Future US development would benefit from addressing more resources for up-to-date equipment as well as standardized education plans.

### Electronic supplementary material

Below is the link to the electronic supplementary material.

Supplementary Material 1

Supplementary Material 2

## Data Availability

The datasets used and analyzed during the current study is available from the corresponding author upon reasonable request.

## Abbrevations

US: Ultrasound
