# Supplementary material for: Pediatric ultrasound practice in Italy: an exploratory survey
Source: Ital J Pediatr. 2024 Jun 9;50:114. doi: 10.1186/s13052-024-01680-3 (PMC11163714; doi:10.1186/s13052-024-01680-3)
Supplement: Supplementary file 1 — Supplementary Material 1 [file 13052_2024_1680_MOESM1_ESM.docx]

Survey Questions

First name initial:

Last name initial:

1. Age:
2. 31-40
3. 41-50
4. 51-60
5. >60
6. What gender do you identify with?
7. Male
8. Female
9. To which of the following categories do you belong to?
10. Pediatrician
11. Pediatrics Resident
12. Other (specify)
13. In which Italian region do you work?
14. Abruzzo
15. Aosta Valley
16. Apulia
17. Basilicata
18. Calabria
19. Campania
20. Emilia-Romagna
21. Friuli-Venezia Giulia
22. Lazio
23. Liguria
24. Lombardy
25. Marche
26. Molise
27. Piedmont
28. Sardinia
29. Sicily
30. Trentino – South Tyrol
31. Tuscany
32. Umbria
33. Veneto
34. What type of facility do you work in?
35. Family pediatrician
36. Hospital
37. Private Hospital
38. University Hospital
39. Other (specify)
40. What department do you work in?
41. General pediatric unit
42. Pediatric ED
43. Neonatology unit
44. Pediatric consultant in adult ED
45. Outpatient clinic
46. Other (specify)
47. Do you have ultrasound available in your work setting?
48. Yes
49. No
50. What type of ultrasound machine do you have?
51. Cart-based
52. Portable
53. Handheld
54. What type of probe do you have available?
55. Convex
56. Microconvex
57. Linear
58. Sectorial
59. None of the above
60. How many years ago was your ultrasound machine purchased?
61. <1
62. 1 - 5
63. 6 - 10
64. >10
65. Unknown
66. Do you use ultrasound in your work?
67. Yes
68. No
69. How many years of experience in ultrasound do you have?
70. <1
71. 2-5
72. 6-10
73. >10
74. What type of clinical ultrasound you perform in your work
75. Abdomen
76. Hips for screening
77. Heart
78. Musculoskeletal
79. Pelvis
80. POCUS
81. Lung
82. Invasive procedures
83. Kidneys and urinary tract
84. Cranial surface
85. Testicle
86. Thyroid
87. Transfontanellar
88. Other
89. How many scans do you perform per month?
90. <10
91. 11-20
92. >20
93. Do you happen to use clinical ultrasound in the emergency room?
94. Yes
95. No
96. What type of patients do you use clinical ultrasound on in the emergency room?
97. suspected pneumonia
98. respiratory distress
99. trauma
100. decompensation
101. differential diagnosis of shock
102. other (specify)
103. What training did you undergo to practice ultrasound?
104. Lectures with practical session
105. Advanced training course
106. Online course
107. On-site experience
108. Other (specify)
109. Do you have a certification to perform ultrasound scans?
110. Yes
111. No
112. Accredited by:
113. How relevant do you consider the use of ultrasound in guiding your clinical choices?
114. Very relevant
115. Fairly relevant
116. Not very relevant
117. Do you think ultrasound can be useful in clinical practice?
118. Yes
119. No
120. What do you consider to be the main difficulty in using ultrasound?
121. Unavailability of the ultrasound scanner
122. Lack of a well-defined training course
123. Legal liability
124. Non-cooperation of colleagues
125. Other (specify)
126. Do you think that the SarsCov2 pandemic has implemented the use of ultrasound in your field of work?
127. Quite a bit
128. Very much
129. Slightly
130. No
131. Do you think the SarsCov2 pandemic has increased your knowledge of ultrasound?
132. Quite a bit
133. Very much
134. Slightly
135. No

Extra questions for Residents

1. Is ultrasound included in the program of your course of studies?
2. Yes
3. No
4. If not, do you consider the inclusion of ultrasound in the program desirable?
5. Yes
6. No
7. Have you been independently trained to perform ultrasound scans? If so, specify what kind of training you have undertaken:
