# Supplementary material for: Pediatric ultrasound practice in Italy: an exploratory survey
Source: Ital J Pediatr. 2024 Jun 9;50:114. doi: 10.1186/s13052-024-01680-3 (PMC11163714; doi:10.1186/s13052-024-01680-3)
Supplement: Supplementary file 2 — Supplementary Material 2 [file 13052_2024_1680_MOESM2_ESM.docx]

**Reviewer #1:**I appreciate the opportunity to review the manuscript titled "State of the Art on Pediatric Ultrasound Practice in Italy: A National Survey." While the topic is certainly relevant and holds potential for exploring future areas of intervention in terms of training, I have several major concerns about the manuscript that, in my opinion, hinder its publication. There are numerous errors that detract from the clarity of the message and need to be addressed before the manuscript can be considered for publication. Below are my comments and suggestions for improvement:

Terminology and Clarity:

The manuscript should distinguish between "point-of-care ultrasound" and "bedside ultrasound" as they have different connotations and applications in the medical field. It appears that you are describing the use of ultrasound by clinicians rather than radiologists, which is better suited to the term "point-of-care ultrasound."

Thank you for your suggestion, we have replaced the term “bedside ultrasound” with “point-of-care ultrasound” within the manuscript.

Introduction:

Clarify the term "pediatric practitioners." Specify whether you are referring to pediatric consultants or attendings in the UK or the US healthcare systems.
Thank you. The term “pediatric practitioners” is referred to pediatric consultants so we have modified the sentence.

What do you mean with current state of US techniques? Are you willing to describe which are the techniques mostly used by clinicians?

We apologize for the lack of clarity about our intent. The aim of the survey is to investigate the use of US among pediatricians and pediatric residents and, if so, to describe the characteristics of the US machines, the number of scans carried out, the years of experience of the performers and the main US applications. Therefore we have better specified the aim of the survey in the introduction section.

Materials:

Line 61: Explain why pediatric trainees were included in the study, as this inclusion might not be typical for such surveys.

Thank you for this observation. In Italy residents have both training and work contract and they provide great support to the pediatric consultant’s practice. Therefore we decided to include them in the survey.

Lines 17 onward: Consider that describing all the possibilities of each domain is redundant, as you plan to describe them in the results section.

We completely agree whit you about this point. We decided, according also to other reviewer’s request, to add the full version of the questionnaire as annex.

Materials (Anatomic Region Scanned):

The distinction between scanning by applications and by anatomical regions is unclear and should be clarified.

We meant to list different US applications so we replaced the term “anatomical regions” into “US applications”.

The use of a 4-point Likert scale should be justified, as it is essentially a forced scale with no neutral option. Discuss why this choice was made.

We know that, usually, Likert scales are odd-numbered scales. We decided to adopt a 4-point Likert scale in order to obtain specific responses on US practice.

Results:

Address the response rate, as it is essential to assess the survey's representativeness.

The response rate is 11%. We have added this information in the text.

Explain if any measures were taken to prevent respondents from submitting multiple answers. Multiple answers were possible only for “setting” and “type of probe”. For all the other characteristics, participants were directed to answer just one answer.

Table 1: Consider ordering age groups, and reconsider including certain categories like retired pediatricians, radiologists, neonatologists and cardiologists.

Thank you for your advice. We rearranged the participants in order of age. We decided to report all the categories who answered the survey and who are registered with the Italian Society of Pediatrics.

Clarify the distinction between hospital types, such as pediatric, university, and general hospitals.

Thank you for this observation. University hospital is referred to a teaching hospital while general hospital is referred to community/district hospital. We included Pediatric Hospital in University/teaching Hospital. We added these distinctions in Table 1.

Correct the reference to "pediatric consultant in adult ED" to "Adult ED."

We have replaced "pediatric consultant in adult ED" with "Adult ED" in Table 1.

The "other" category account for a number of NICU and PICU specialists who are more trained in US and 6 pediatricians who retired. Try to explain why these were included.

We included these special categories because they are registered with the Italian Society of Pediatrics.

Table 2 (Anatomical Regions Scanned):
Clarify the meaning of "general" US application.

Thank you for the suggestion. We deleted table 2 and created three new graphs.

Address overlapping age ranges (1-5 and 5-10) and consider the need for clearer categories.

We apologize for the error in the text. The correct groups are <1, 1-5, 6-10, >10 so we have modified the table.

Specify that "Hips for screening," "Heart," and "MSK" are applications rather than anatomical regions.

Thank you for the suggestion. We created a new graph (Graph 1) entitled “US applications”.

Consider reclassifying "vascular access" under procedural ultrasound or invasive procedures.

We added "vascular access" to the invasive procedures group in Graph 1.

There's no reason to put a POCUS group in the anatomical region scanned. POCUS is a modality of use of US by clinicians.

We agree with your observation, so we have created a new graph (Graph 1) entitled “US applications”.

Results (Pediatric Resident vs. Trainees):

Maintain consistent terminology throughout the manuscript regarding categories like "pediatric resident" and "trainees."

We thank you for your suggestion. We intended to use the term “pediatric resident” as a synonymous of “trainees” but for clarity purpose, we replaced the term “trainees” with “pediatric residents” throughout the manuscript.

Results (Echography):

Clarify the usage of "echography" alongside terms like "US" and "POCUS" to avoid confusion for readers. Thank you for the suggestion. We replaced the term “echography” with “US” in order to avoid confusion for the readers.

Results (Shock vs. Hemodynamic Decompensation):

Define the difference between "shock" and "hemodynamic decompensation" if relevant to your study. FOCUS can rapidly assess global cardiac function and can help distinguish between the different types of shock. Due to the similar meaning of terms "shock" and "hemodynamic decompensation", which is possibly confounding for readers, we decided to unify them under a single group called “undifferentiated shock”.

Results (Family Pediatricians):

Provide an explanation and percentages for the statement regarding the use of US by family pediatricians.

Thanks. We added a possible explanation about high percentages of family pediatricians performing US in the discussion section.

Table 4 (Advanced Training Course):

Define what "advanced training course" entails in terms of education or skill development.

Thank you. AS “advanced training course” we meant an evolving application course for those who wanted to deepen and further develop specific US skills.

Discussion:

Reevaluate the relevance of the discussion on COVID-19 to the goals of the survey.

Thanks for this observation. We carried out a further analysis for pediatricians working in the emergency department or not. We reported the results and discussed.

Discussion (Ultrasound Spread):

Acknowledge the importance of education alongside equipment availability in the spread of ultrasound. The spread of US is connected to the availability of US machines. You missed to mention that there's another important factor, education. Ideally both these factors should come together for a well-structured implementation of bedside ultrasound. Having a machine without proper education doesn't allow to set a program at any level. (check ACEP statement-guideline)
Thanks for this observation. We stressed out the importance of education in the discussion section.

Discussion (Credentialing):

Remove discussions related to "credentialing" unless supported by appropriate survey questions.

Thanks. Question 19 asks for US certification.

In conclusion, this manuscript requires significant revisions to address the outlined concerns and improve its clarity and relevance. I recommend enlisting the help of a native English speaker and a medical writing expert to assist in the revisions. Once these issues are resolved, the manuscript may be suitable for publication.

**Reviewer #2:**I have read with interest the paper "State of the art on Pediatric Ultrasound Practice in Italy: a national survey " as US integration should be considered a useful technical integration/evolution of the clinical exam. The paper reports a survey on the US practice among paediatrician of the Italian Society of Pediatrics. It is an interesting topic but it should be improved and enriched before to be considered for publication.
Here my commentaries:
Title
The title seems to refer to a wider and much more exhaustive study. I agree with the Author that the survey has been performed including paediatricians from all over Italy, and thus it has a national dimension, but I am not sure that 1098 paediatricians could reflect properly the current state of US in a complex and varied reality as the Italian is. Please reconsider the title, in order to properly describe the paper.

Thank you for you suggestion. We have changed the title in “Pediatric Ultrasound Practice in Italy: an exploratory survey”.

Methods
1-      The main purpose of a survey is to give the reader a "general view" of a specific topic. The Author included a varied scenario of paediatricians from private and public hospitals, including full-private practice and University Hospitals, and it may concur to improve the quality of the paper. Nevertheless there are two main criticism in methodology:
a.      only paediatricians of the I.S. Pediatrics have been included;

Thank you for the observation. We chose to include members of the Italian Society of Pediatrics in the study in order to reach as many participants as possible through a mailing list.

b.      as reported by the Author itself, paediatricians have been included on voluntary basis, and thus, a particular interest in US practice could be expected among those included in the study.
This approach is already a selection itself and it could results in a selection bias. This aspect should be more stressed in the discussion and limits sections, as the "general view" on the topic could be influenced by this approach.

Thank you. We already added this limitation in the discussion section but we will stress this point.

2-      The questionnaire should be added to the paper in both its forms

Thank you for this suggestion. We added the questionnaire as an annex to the paper.

Results
1-      The Author reports a descriptive table showing the distribution of paediatricians among public, private activity or hospitals. Each of these areas has specific needs, limits and characteristics, which can also change according to the size of the hospital and the location in the national territory. The Author should report differences in US practice according to the hospital (university or not, public or private, central or peripheral) and to practice (public or private), with a proper statistical analysis, in order to underline significant differences. This approach could provide further interesting data to be discussed in the discussion section, and strategies for improvement of the US training.

We have added tables with the subdivision between public and private structures and between university and non-university structures. There is no statistical difference between the different populations analyzed.
2-      Differences in US practice should be reported also according to the working dept. and to the national area. As said above, these information could give important data on when, how and where the improvement of US training can offer better results, and should be discussed in the discussion.

No statistically significant differences were found by dividing the sample by geographical area (north, central or south) or by working department. We have added this evaluation in the results.

3-      The Author states "Nearly one third of respondents (42.8%, n=330) assessed that US machines had been provided from 1 to 5 years prior to the survey":  42.8% is much more than one third (33%), this sentence should be corrected. To this point it is unclear if the Author refers to a first assignment of a US machine or whether it refers to the in use one at the department (have some US machine been substituted?). It is an important difference as the Author states in the discussion section that "US practice seems to be relatively recent tool in pediatrics [4], considering that machines have been provided mostly 1-5 years prior to the survey".

We replaced the sentence within the manuscript reporting just the percentage.

We refer to the US machine in use within the department at the time of the survey.

4-      I noticed that more than 95% of family paediatricians reports a routine use of US in common practice. The US practice seems to be much more common among  family paediatricians that among hospital ones; and it is in contrast with the common experience. Could it be due to a bias in selection? The Author should comment on it.

Yes, we believe that this result probably reflects a bias selection. We added a comment on it in the discussion section.

Discussion
1)      Page 7, lines 7-24. This part of the discussion should be strongly improved if the Author could report and comment the differences on US practice according to the working department, region and public/private/University Hospital of paediatrician/residents.

Thanks for the suggestion. We improve this part of the discussion reporting new data obtained.

2)      Page 11, lines 46-58. During the early phase of the COVID pandemic the Health Service and the assistance were focused only on emergency patients and COVID patients, so I am not surprised that during the COVID pandemic the use of US have not been improved or enlarged in general. The Author should, to this point, focus the attention on ED paediatricians, as in this population an improvement of US practice could be shown during the pandemic (lung/thorax seems to be the most frequently studied district).
Thanks for the suggestion. We have divided the population into two groups (working or not in ED) and we have seen that those working in ED have increased the use of ultrasound and knowledge during the Covid pandemic. We added the data in the results and then discussed.

Conclusions
1.      "To date, the unavailability of US machine and the lack of specific training programs are perceived to be main barriers for US application". This is an interesting conclusion and should be commented  according to territorial differences and working dept., in order to underline specific needs and differences.
Thank you. We were not able to point out specific needs or differences through this survey as we did not find significant data. We aim to increase the sample in the future in order to carry out a further analysis.

**Reviewer #3**: Dear authors,

I consider that your research is interesting but it must provide the following improvements in order to be published

Title- You should improve the title to make the study seem more attractive. And indicate the type of descriptive study, remove "national survey"

Thank you for you suggestion. We have changed the title in “Pediatric Ultrasound Practice in Italy: an exploratory survey”

Introduction line 49 I think you can specify your specific objectives more.

Thank you. We better reported the objectives of the survey.

Furthermore, this section is too short, I am sure that in the research you will find references in other articles about setting, the training needs as well as the difficulties of implementing US. Furthermore, it is interesting that it indicates other possible issues that are identified in other countries even though the survey is in Italy.

Thank you for this observation, we implemented the introduction with other issues regarding US practice.

Material and methods
I would like you to indicate if there is another study in the research with the characteristics of the survey they carried out. Line 7 and 8. Also, on this line, also indicate if there are other studies that have not used unsigned consent and refer to it. I believe that a higher methodological quality of the study would imply the non-anonymity of the survey but with unsigned consent.

The consent was implied through survey participation. Participation in the survey was voluntary. Identifiable information was not obtained to maintain anonymity (participants were asked to register their initials prior to start).

- Parri N, Berant R, Giacalone M, Jones SD, Friedman N; REPEM POCUS collaboration. Dissemination and Use of Point-of-Care Ultrasound by Pediatricians in Europe: A Research in European Pediatric Emergency Medicine Network Collaborative Survey. Pediatr Emerg Care. 2022;38:e1594-1600.
- Eisen LA, Leung S, Gallagher AE, Kvetan V. Barriers to ultrasound training in critical care medicine fellowships: a survey of program directors. Crit Care Med. 2010 Oct;38(10):1978-83.
- Marin JR, Zuckerbraun NS, Kahn JM. Use of emergency ultrasound in United States pediatric emergency medicine fellowship programs in 2011. J Ultrasound Med. 2012;31:1357-63.
- Arichai P, Delaney M, Slamowitz A, Rosario R, Gordish-Dressman H, Basu S, et al. Pediatric Residency Point-of-Care Ultrasound Training Needs Assessment and Educational Intervention. Cureus. 2022;14:e28696.

Provide the total questionnaire as an annex and cite it in this section

Thank you for this suggestion. We added the questionnaire as an annex to the paper.

Make subheadings in this section:
- Subjects. Make a table with the sociodemographic variables that are indicated in the results section but are cited in this subsection. This table must separate the variables by sex.

Thank you for this suggestion. We have reported the sociodemographic variables in Table 1 in the results’ section.

- Questionnaire. Indicate if the questions were randomized.

The questionnaire was not randomized.

Results
I consider that most of the stables can better see their results in graphs.

Thank you. We eliminated table 2 and created 3 graphs.
Line 55 is better called graph than figure 1.

Thank you, we replace the term “figure” with “graph” as suggested.

Add a legend to figure 1.

We have added “Graph 1. Limitations to pediatric US application. Data are expressed as a percentage”.
Separate the results by sex in each table, you will obtain greater analysis

Thanks for the suggestion. We tried to divide the sample based on sex but there were no statistically significant so we decided not to report in the text.

Discussion
Please divide the discussion into sections of each objective explained in the introduction.
Also indicate in each result if there are studies where these results can be compared with those of other countries if they exist in the research.
Consider whether use in certain areas may be influenced by the need for higher resolution of the US device
Thank you for these observations. We divided the discussion into sections as requested. We also added some comparisons for each section of the discussion, depending of available literature.

Conclusions
State the conclusions in shorter sentences.

Thanks, we modified the sentences by reducing the length of periods.
